# Correlation of Plasma FL Expression with Bone Marrow Irradiation Dose

**DOI:** 10.1371/journal.pone.0058558

**Published:** 2013-03-07

**Authors:** Mary Sproull, Dane Avondoglio, Tamalee Kramp, Uma Shankavaram, Kevin Camphausen

**Affiliations:** Radiation Oncology Branch, National Cancer Institute, Bethesda, Maryland, United States of America; French Blood Institute, France

## Abstract

**Purpose:**

Ablative bone marrow irradiation is an integral part of hematopoietic stem cell transplantation. These treatment regimens are based on classically held models of radiation dose and the bone marrow response. Flt-3 ligand (FL) has been suggested as a marker of hematopoiesis and bone marrow status but the kinetics of its response to bone marrow irradiation has yet to be fully characterized. In the current study, we examine plasma FL response to total body and partial body irradiation in mice and its relationship with irradiation dose, time of collection and pattern of bone marrow exposure.

**Materials/Methods:**

C57BL6 mice received a single whole body or partial body irradiation dose of 1–8 Gy. Plasma was collected by mandibular or cardiac puncture at 24, 48 and 72 hr post-irradiation as well as 1–3 weeks post-irradiation. FL levels were determined via ELISA assay and used to generate two models: a linear regression model and a gated values model correlating plasma FL levels with radiation dose.

**Results:**

At all doses between 1–8 Gy, plasma FL levels were greater than control and the level of FL increased proportionally to the total body irradiation dose. Differences in FL levels were statistically significant at each dose and at all time points. Partial body irradiation of the trunk areas, encompassing the bulk of the hematopoietically active bone marrow, resulted in significantly increased FL levels over control but irradiation of only the head or extremities did not. FL levels were used to generate a dose prediction model for total body irradiation. In a blinded study, the model differentiated mice into dose received cohorts of 1, 4 or 8 Gy based on plasma FL levels at 24 or 72 hrs post-irradiation.

**Conclusion:**

Our findings indicate that plasma FL levels might be used as a marker of hematopoietically active bone marrow and radiation exposure in mice.

## Introduction

Total body irradiation (TBI) used in conjunction with myeloablative chemotherapy remains the clinical standard for many conditioning regimens for bone marrow transplantation. Clinically, TBI exposes all parts of the body to a homogenous irradiation field resulting in a uniform received dose to all organs excepting the lungs which can be shielded. [1] Hematopoietic stem cell transplantation (HSCT), has greatly advanced both in the new types of HSCT now being offered and the sophistication of how these therapies are administered. Given the evolving nature of the field there is a great deal of variation in TBI regimens including dose rate, total dose received as well as single vs. fractionated dose and dosing schedule. As such, there is currently no single established therapy regimen for TBI in HSCT. Early TBI conditioning regimens were based on classical models of bone marrow sensitivity to radiation conducted in animals, and recent TBI dosing regimens are the subject of randomized clinical trials as new TBI therapies are tailored to modern HSCT and to reduce regimen-related toxicity. [2], [3], [4].

*fms*-like tyrosine kinase (Flt3) ligand (FL) is a cytokine known to induce proliferation and differentiation of bone marrow stem cells of myeloid and lymphoid origin. [5] It is a transmembrane protein that is biologically active both in its bound and soluble forms. FL is primarily expressed in the bone marrow though FL mRNA is found in a wide variety of tissues. [6], [7], [8] In patients undergoing fractionated radiotherapy, a direct correlation has been made between plasma FL levels and the percentage of irradiated bone marrow. [9] An increase in plasma FL levels has also been correlated with a decrease in WBC count in patients undergoing stem cell transplantation. [10] In several recent case reports where individuals were accidently exposed to radiation, FL was used to monitor hematopoietic aplasia and FL levels appeared to correlate with the amount of radiation exposure. [11], [12], [13].

To assess the utility of plasma FL as a potential marker of bone marrow tissue response in TBI conditioning regimens, the dynamics of FL expression were examined over varying doses and time points of collection post total body irradiation. These FL levels were used to create a predictive model to estimate radiation dose. This model was then examined for its ability to quantify unknown radiation dose in a blinded setting based on plasma FL levels. We also examined the relationship between FL expression and partial body irradiation exposures in order to clarify whether the plasma FL response to irradiated bone marrow is a uniform or heterogeneous response.

## Materials and Methods

### Animal Model

All animal studies were conducted in accordance with the principles and procedures outlined in the NIH Guide for the Care and Use of Animals and procedures were approved by the NIH Lab Animal Safety Program under Protocol #ROB-135. Ten week old female C57BL6 mice received a single whole body or partial body irradiation dose of 1–8 Gy or sham radiation from a Pantak X-ray source at a dose rate of 2.28 Gy/min and all group cohorts were comprised of between 3–10 animals. Plasma was collected by mandibular or cardiac puncture at 24, 48 and 72 hr post-IR as well as 1–3 weeks post-irradiation in Lithium Heparin blood collection tubes (BD Biosciences). Blood samples were spun at 10,000 RCF for 10 minutes at room temperature and stored at -80°C. As described in previous studies analyzing specimen handling variables, several processing variables for FL were examined and are shown in Table S1. [14], [15].

### Mouse FL Measurement

Flt3-ligand (FL) levels were determined in mouse plasma using a commercially available ELISA kit (R&D Systems) according to the manufacturer’s instructions.

### Statistical Analysis

*In vivo* experiments were analyzed independently of each other and unless otherwise indicated the data presented are specific to each individual animal. Statistical analysis of FL values was done using a Student's *t* test and a probability level of *p*<0.05 was considered significant. Data is presented as mean ± SEM. All FL values were corrected for interplate variation. The Linear regression model and Gated values model of dose prediction at 24 and 72 hr post-IR were generated using FL values from animal experiments encompassing 66 control and 109 TBI irradiated mice. A linear regression equation was generated using the averaged FL values at 1 Gy, 4 Gy and 8 Gy, at either 24 hr or 72 hr post-IR. Using known FL pg/ml/irradiation dose matched values; FL gates were also used as guidelines for predicting unknown dose. For this method FL pg/ml “gates” were generated using one standard deviation above/below the pooled average with adjustments to accommodate the lowest false positive rate. To further reduce our rate of error we attempted to account for outliers within groups by examining the 24 hr/72 hr post-IR FL trend. Samples were taken serially from the same mouse by mandibular stick at 24 hr and cardiac stick at 72 hr post-irradiation at doses of 1, 4 and 8 Gy. If FL levels remained constant between the two time points the exposure was predicted to be 1 Gy or less, but if FL increased from 24 hr to 72 hr the mouse received a dose equal to or greater than 4 Gy. Analysis of the 24 hr/72 hr FL trend was used to validate both the linear regression and gated dose prediction methods at 72 hr post-IR. Blinded FL values were evaluated by either of these methods at 24 and 72 hr post-irradiation. Mice were grouped into either control, 1, 4, 8 Gy groups or control/1 Gy, 4/8 Gy groups. For this study, we required a dose prediction method to accurately predict >70% of the samples to be deemed successful and all methods of dose prediction were done blinded.

## Results

### Elevated Plasma FL Levels in TBI Irradiated C57BL6 Mice

**Figure 1:**
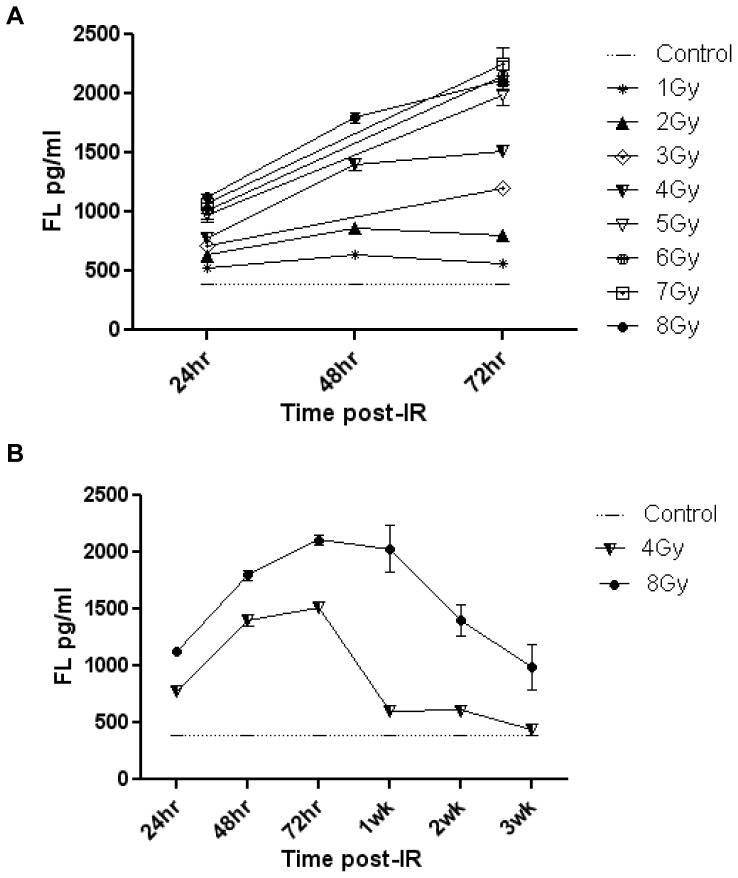
FL Levels Following TBI. FL levels correlate with dose of irradiation and time of collection. A) Plasma FL levels in TBI C57BL6 mice at 24, 48 and 72 hrs post-IR at doses between 0 and 8 Gy. B) Extended time course of plasma FL expression in TBI C57BL6 mice at 0, 4 and 8 Gy at time points between 24 hrs and 3wks post-IR. Values reflect the mean ± SEM.

Following irradiation there was an increase in FL levels in the plasma of total body irradiated (TBI) C57BL6 mice. This increase was seen at 24, 48 and 72 hours post-irradiation at doses of irradiation between 1 and 8 Gy. FL levels from mice exposed to 1 Gy were significantly higher (*p*<.001) than the un-irradiated control mice at 24, 48 and 72 hours post-irradiation (Fig. 1A). Mice in all dose groups between 2 and 8 Gy had FL values similarly significant over control (p<.001) (Fig. 1A). At the higher doses of irradiation, FL levels continued to rise at 48 and 72 hrs but at lower doses of 1 and 2 Gy the levels started to return to baseline at 72 hrs post-IR.

**Figure 2:**
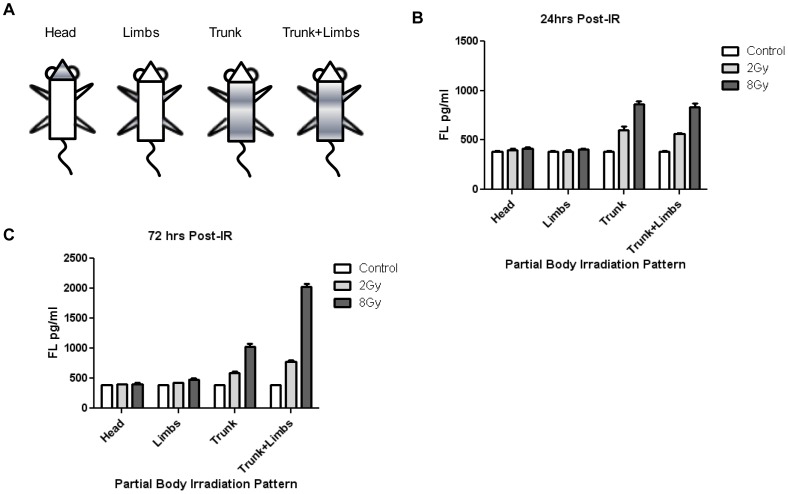
Partial Body Irradiation Patterns and FL Response. Plasma FL expression following heterogeneous partial body irradiation patterns in C57BL6 mice. A) Mice were partially shielded to irradiation using the represented partial body diagrams with either the head, limbs, trunk, or trunk+limbs exposed. B) Plasma FL expression in partial body exposed mice at 0, 2, and 8 Gy at 24 hrs post-IR. C) Plasma FL expression in partial body exposed mice at 0, 2, and 8 Gy at 72 hrs post-IR. Values reflect the mean ± SEM.

To evaluate the duration of FL elevation following irradiation we measured plasma FL at 0, 4 and 8 Gy at the later time points of 1–3 weeks post-irradiation. For both the 4 Gy and 8 Gy exposures the peak elevation of FL was seen at 72 hrs. At 4 Gy, 72 hr post-IR FL was significantly higher than control but decreased at 1 week and 2 weeks post-irradiation returning to near control levels by week 3 post-irradiation (Fig. 1B). 8 Gy 72 hr post-IR was higher than control with serially decreasing values at the 1 week, 2 week and 3 week post-irradiation time points (Fig. 2B). All FL values at 4 Gy and 8 Gy were significantly higher than control with *p* values of (*p*<.001) except for 4 Gy and 8 Gy week 3 which had *p* values of (*p* = .04, *p* = .02) respectively. (Fig. 1B).

### Elevated Plasma FL Levels in Partial Body Irradiated C57BL6 Mice

To further examine the relationship of FL and irradiation of bone marrow, several partial body irradiation patterns were examined by isolating either the more hematopoietically active bone marrow of the sternum, ribs, pelvis and vertebrae for exposure, or other less hematopoietically active sites such as the limbs or head. (Fig. 2A) Using these partial body-irradiation profiles, an increase in FL over control was seen at 2 Gy and 8 Gy, 24 hrs post-irradiation with exposure to the trunk area alone or exposure to both trunk and limbs while shielding the head, but not with exposure to only the head or only the limbs. (Fig. 2B) This FL increase was significant at 2 Gy with *p* values of (*p*<.02, *p*<.001) and at 8 Gy with *p* values of (*p*<.01, *p*<.01) respectively for irradiation of trunk only and trunk and limbs only. A similar FL increase post-irradiation was seen at 72 hrs with both 2 Gy and 8 Gy but only with irradiation of the trunk only or trunk and limbs together. (Fig. 2C) Excepting irradiation of trunk only at 2 Gy which had a (*p*<.02) all of the trunk area irradiation patterns at 72 hrs had *p* values of (*p*<.01) and were significantly higher than control. With irradiation of the trunk and limbs together, no significant difference was seen in FL values over irradiation of the trunk alone at 24 hrs but a significant difference was seen at 72 hrs at 2 Gy and 8 Gy (*p*<.001). With irradiation of only the head or only the limbs no significant increase of FL over control was seen at 72 hrs.

### Blind Study Dose Prediction Results

**Figure 3:**
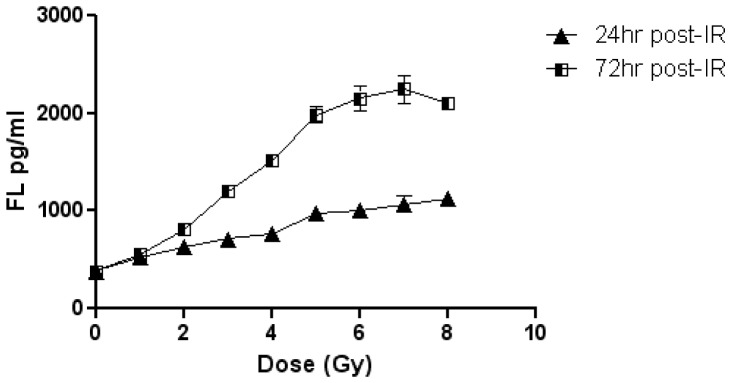
FL Blinded Dose Prediction Model. FL values at doses of 0–8 Gy in TBI C57BL6 mouse plasma used to generate dose prediction models for blinded dose prediction study at 24 and 72 hrs post-IR. Values reflect the mean ± SEM.

To test whether FL is a useful indicator of received dose to the bone marrow, a linear regression model and gated dose prediction model were generated for blinded dose prediction using previously determined plasma FL values at 24 and 72 hrs. (Fig. 3) At 24 hr there was not enough discrimination between the Control and 1 Gy FL values and the 4 Gy and 8 Gy values to accurately predict the majority of the samples correctly. But when the dose groups were analyzed as Control/1 Gy and 4 Gy/8 Gy there was consistent dose prediction. At 72 hr however, samples were able to be accurately identified in all of the respective groups of Control, 1, 4 and 8 Gy.

Analysis of plasma FL levels at 24 hr post-IR using the gated dose prediction method correctly predicted 18/18 mice from the Control/1 Gy group and 16/19 mice in the 4 Gy/8 Gy group. The linear regression method was similarly accurate correctly predicting 17/18 mice from the Control/1 Gy group and 17/19 mice in the 4 Gy/8 Gy group. At 24 hr post-IR, using either dose prediction method, 92% of the samples were correctly grouped into Control/1 Gy or 4 Gy/8 Gy (Table 1).

At 72 hr post-IR, the gated dose prediction method correctly predicted 8/10 Control mice, 6/8 mice who received 1 Gy, and 8/9 4 Gy and 9/10 8 Gy. The linear regression method was slightly lower with 8/10 correct for Control and 6/8 1 Gy, 7/9 4 Gy and 9/10 8 Gy correct. Overall the gated prediction method at 72 hr post-IR correctly predicted dose with 84% accuracy and the linear regression with 81% (Table 1). Using either method of analysis plasma FL in mice demonstrates a high degree of accuracy predicting irradiation dose at 24 and 72 hrs post-irradiation.

**Table 1:** Results of Blinded Dose Prediction Analysis Using Plasma FL at 24 hrs and 72 hrs following Total Body Irradiation.

Treatment Group	Prediction Method		% Correct	# Correctα	Collection Timepoint
	Gated	Linear			
Control/1 Gyβ	x		100	18/18	24 hr
		x	94	17/18	24 hr
4 Gy/8 Gyβ	x		84	16/19	24 hr
		x	90	17/19	24 hr
Control	x		80	8/10	72 hr
		x	80	8/10	72 hr
1 Gy	x		75	6/8	72 hr
		x	75	6/8	72 hr
4 Gy	x		89	8/9	72 hr
		x	78	7/9	72 hr
8 Gy	x		90	9/10	72 hr
		x	90	9/10	72 hr

α #Correct represents the number of correct predictions over # of total animals in that group. 72 hr time point prediction analysis includes evaluation of 24 hr/72 hr FL trend as described in the text.

β Samples pooled from the stated treatment groups and evaluated as a single cohort.

## Discussion

### Applications in the Clinical Setting

Hematopoietic stem cell transplantation (HSCT) is defined as the replenishment of the hematopoietic stem cell compartment using stem cells derived from bone marrow, peripheral blood or cord blood. These grafts may be autologous or allogenic from matched related or unrelated donors and the preparative regimen for many of these procedures includes some form of total body irradiation (TBI). [2], [16] TBI has advantages over myeloablative chemotherapeutic regimens alone as the exposure to a uniform field of radiation ensures that “sanctuary sites” such as the testes or less vascularized areas of the bone marrow niche are purged. TBI cannot be used alone however as the doses required to ensure complete bone marrow depletion followed by new stem cell engraftment are too toxic and as such lower TBI doses must be used in combination with chemotherapy treatment. [2] TBI schedules for bone marrow ablation follow a variety of protocols with variation to dose rate, total received dose and single vs. fractionated treatments. These preparative regimens are continually modified as newer TBI treatment protocols are developed in order to improve therapeutic outcome and reduce treatment toxicity. [4], [17], [18], [19], [20] A validated marker of irradiated bone marrow might be useful in the designing of future TBI preparative regimens potentially increasing the efficacy of treatment and allowing for the modification of treatment to the individual patient.

FL is a cytokine known to induce proliferation and differentiation of hematopoietic progenitor cells and levels of FL in the serum have been shown to have an inverse correlation to number and proliferative potential of hematopoietic precursors. Elevation of FL is seen in response to a variety of stresses to the hematopoietic stem cell niche including hematologic diseases such as congenital anemia and acquired aplastic anemia induced by chemotherapy. [21] In diseases affecting only single blood lineages, an increase in FL is not seen. [22] Though FL mRNA is ubiquitously expressed throughout the body, the protein has only been found on T lymphocytes and stromal fibroblasts within the bone marrow microenvironment. [21], [22], 23 Exposure to radiation of the hematopoietic stem cell niche also results in an increase to FL expression. This has been seen in patients undergoing radiation therapy with simultaneous onset of leucopenia, neutropenia and thrombocytopenia. [9], [10], [24].

Though preliminary studies have linked FL expression with irradiated bone marrow the dynamics of plasma FL expression with regard to radiation dose, kinetics of expression post-irradiation and pattern of exposure have not been fully characterized. In our study, a range of clinically relevant radiation exposures and time points of collection were selected to explore the dynamics of FL expression and then validated in a blinded study to generate a working model of FL as a marker for irradiation of bone marrow.

Using two distinct methods of statistical analysis, total body irradiated mice were correctly identified into dose received cohorts based on plasma FL levels at 24 and 72 hrs post-irradiation. As there is up to a 4-fold difference in cytokine expression following irradiation between individual mice of the same strain, the FL trend over various time points post-IR was examined and proved helpful in identifying dose response groups and may be useful in compensating for potential variation in FL expression. [25] Such variation may be prevalent in human samples as differences in human FL baseline levels have been shown to vary with race and over geographic region. [13] Though in one study patients undergoing fractionated radiotherapy showed little variation in baseline FL levels amongst men and women between 20 and 60 years of age. [9].

The kinetics of FL elevation following TBI exposure was examined at serial time points post-irradiation. FL increase correlated with both dose and time post-irradiation peaking at 72 hr with subsequent decrease back to near baseline at week 3 post-IR. The initial rise in FL values following IR is attributed to loss of bone marrow function and the later return of FL over time to normal values with recovery of the hematopoietic stem cell population. [9], [10] This FL trend is similar to that seen in patients with accidental radiation exposures and subsequent peripheral lymphocyte recovery. [11], [12] Though it should be noted that the time points of 24, 48 and 72 hrs used in this study were chosen to reflect the trend of FL kinetics in response to radiation in mice and that this timeframe may not correlate literally to humans.

The FL response to TBI appears to directly relate degree of FL response with the amount of radiation received. This relationship has also been expanded to partial body radiation exposures relating FL expression with the percentage of irradiated bone marrow. [9], [26] One study examined this idea using partial body exposure patterns by irradiating mice along the sagittal and transverse axis of the trunk area in effect, dividing the total exposed bone marrow into quarters. Using this bone marrow exposure pattern increasing FL expression was correlated with increasing area of irradiated bone marrow. [27] Though these data suggest that the relationship between plasma FL expression and irradiated bone marrow applies equally to all bone marrow, this assumes all bone marrow is equally responsive to irradiation. We postulated that FL as a marker of hematopoiesis is predominantly reflective of only hematopoietically active bone marrow and not total bone marrow. To this end, we examined several partial body irradiation patterns which separated classically accepted centers of hematopoiesis such as the vertebral column, sternum and pelvis from the remaining areas of the skeleton. Using this model, increases in FL expression were seen only with irradiation of the trunk area and not with irradiation of the head or limbs. This clarifies the effect seen in the previous study where the exposure pattern quartering the mouse along the midline effectively divided the trunk area into equal parts, essentially fractioning the hematopoietic centers as well. Though the classically held centers of active hematopoiesis in the adult human remain the pelvis, vertebral column, sternum and ribs, cranium and the epiphyseal ends of the femur and humerus, bones with the highest percentage of red marrow are actually the pelvis, vertebrae, sternum and ribs. [28], [29].

This is not to say there is not hematopoietically active bone marrow in the limbs or cranium as the femur is the common site of stem cell harvesting in mice and one patient study has shown that sufficient number of stem cells for successful autologous stem cell rescue can be isolated from a bone flap during craniotomy. [30], [31], [32] In the mouse, all bones support hematopoiesis in addition to the spleen though the greatest concentrations of red marrow are found in the cranium, lower limbs, spine and pelvis. [33], [34].

Despite these differences in areas of highest stem cell activity in the mouse and human stem cell niche, the ability of the hematopoietic system to repopulate stem cells from residual unaffected populations remains a conserved function in both species. [27], [35] We also saw that the FL response following irradiation to only the trunk was significantly higher than control for both 2 Gy and 8 Gy at 72 hrs post-IR, but less than the FL expression of irradiation of the trunk and limbs together at the same time point and dose. This suggests that though irradiation of the limbs alone was not significant over control, when the limbs were irradiated together with the trunk there was an additive effect and that the bone marrow of the limbs may contribute to the FL response when irradiated in concert with the more active hematopoietic bone marrow of the trunk. We did not see an additive effect of including the head in the exposure field as irradiation of the trunk and limbs had FL values similar to TBI values at the same doses and time points post-IR. It may also be that with irradiation of only the limbs or only the head, enough of the hematopoietically active stem cell niche was spared to repopulate the irradiated areas without the need for increased expression of FL. These partial body irradiation data suggest that FL is a marker not of irradiated bone marrow, but of radiation induced damage to the hematopoietically active bone marrow system.

### Applications as a Radiological Biodosimeter

There is an emerging body of evidence linking FL expression with bone marrow status and radiation exposure. The data here show a direct relationship between FL and TBI dose. This radiation dose dependent expression of FL may have applications for use as a radiation biodosimeter. In the event of a radiological or nuclear bioterrorist incident, it is expected that mass population screenings will be needed to triage exposed individuals. Determining the level of radiation exposure is critical for identifying those individuals who best benefit from medical intervention as TBI exposures of 2–10 Gy experience increased survival with treatment but those with <1 Gy do not require treatment to survive and there are no current treatments ensuring survival of >10 Gy exposures. [36], [37], [38] A biodosimeter would also be helpful with the infrequent but difficult to quantify accidental exposures that arise from lost radioactive sources in industry. [11], [12] In such cases, it is exceedingly difficult to determine level of exposure due to variation in irradiation field, heterogeneity of exposure pattern, whether the radiation was TBI or resulting from inhaled airborne particles or ingested from ground contamination. [35], [39].

Treatment of the hematopoietic system is one of the main priorities in management of radiation-induced-multi-organ-failure (RIMOF) of irradiated victims. [40] Bone marrow is one of the early dose-limiting organs due to its high degree of cell turnover. Advanced leucopenia and thrombocytopenia are commonly associated with radiation induced bone marrow damage and are a critical priority in radiation exposure treatment regimens. Early intervention with cytokine therapy or bone marrow transplantation can reverse the effects of radiation induced bone marrow depletion. [40], [41] This makes FL whose expression pattern has been shown to correlate both with radiation dose and bone marrow function an attractive choice as a radiation biomarker. [9], [24] Preliminary data from various studies involving mice, non-human primates and incident reports of human exposure have indicated that FL may be a viable biodosimeter. [11], [12], [24], [27], [42] Yet these findings represent a wide heterogeneity of exposure doses, time points of collection post-IR and species. Moreover these studies used small numbers of test subjects and demonstrate the relationship between FL and irradiation from a proof of concept perspective, not a functioning FL model challenged in a blind sample setting.

Our study demonstrates a quantitative relationship between plasma FL levels and irradiation dose in mice and through validation with a blind study demonstrates a working model of FL as a biodosimeter. This data might be applied to triage for mass screening of an exposed population by first collecting a 24 hr plasma FL sample and stratifying samples into a less than 1 Gy or unexposed group and a greater than or equal to 4 Gy group. Further FL samples from individuals in the 4 Gy group could then be collected at 72 hr post-IR and more definitively classified into 4 Gy or 8 Gy groups. An additional FL sample at 1wk post-IR would also help distinguish the 4 Gy vs 8 Gy groups as 1wk FL values should be significantly lower than 72 hr for those exposed to 4 Gy whereas the 8 Gy exposure 72 hr and 1wk samples would have minimal change. FL also appears to be stable in its plasma soluble form as we noted no significant change in FL levels under several processing variables including time to processing post-venipuncture, type of storage tube, and sub zero storage at either -20C or -80C. (Table S1)) In NHP it has been shown that circadian rhythms do not affect FL expression and we have determined that the psychological stress of being restrained and moderate blood loss do not alter FL in mice. [24] (Data not shown) FL appears to be an ideal candidate as a biomarker for radiation biodosimetry given its dose dependent expression, low intra-individual biologic variability and stability during laboratory analysis and may have practical applications for both mass casualty screenings and therapeutic use in the clinical setting.

### Applications in Future Clinical Protocols

When considering radiation induced morbidity to the hematopoietic bone marrow system it is important to recognize the inhomogeneous distribution of the stem cell niche. [43] The current design of clinical TBI exposures to the hematopoietic system are continuing to evolve as graft failure, recurrence of malignancy and normal tissue toxicity are all related to the conditioning regimen. [3], [44] With the advent of more sophisticated preparatory regimens patient outcomes have been improved by increasing the efficacy of treatments subsequent to TBI, while reducing the side effects of radiation. [19], [45] Reducing the intensity of the conditioning regimen has also allowed patients who may not have been able to withstand radiation induced toxicities of earlier regimens to receive treatment. [46] Plasma FL appears a promising biomarker of radiation induced damage to the hematopoietic stem cell system and might facilitate the tailoring of future conditioning regimens to the individual patient as seen in two pilot studies where FL levels in patients were correlated with hematologic toxicity and radiation dose. [47], [48] The dynamics of FL in response to radiation appears to correlate with dose received, status of hematopoietically active bone marrow and even radioprotection when FL is administered prior to irradiation. [49] FL may have many clinical applications in the design of future conditioning regimens involving radiation.

## Supporting Information

Table S1 **Results of Potential Processing Variables Relevant to Clinical Screening of FL.**
(DOCX)
