# Supplementary material for: Correlation of Plasma FL Expression with Bone Marrow Irradiation Dose
Source: PLoS One. 2013 Mar 7;8(3):e58558. doi: 10.1371/journal.pone.0058558 (PMC3591371; doi:10.1371/journal.pone.0058558)
Supplement: Table S1 — Results of Potential Processing Variables Relevant to Clinical Screening of FL. (DOCX) [file pone.0058558.s001.docx]

Table SI

Results of Potential Processing Variables Relevant to Clinical Screening of FL

| Processing Variable |  | ∆ FL |
| --- | --- | --- |
| Type of Storage Tube* | PPE 1.7 ml Tube | ― |
|  | Nunc 2ml Cryovial | ― |
|  | Corning 2ml Cryovial | ― |
| Subzero Storage** | -80°C / -20°C | ― |
| Time @ 22°C before Processing^δ^ | .5 hrs | ― |
|  | 1 hr | ― |
|  | 2 hr | ― |
|  | 3 hr | ― |
|  | 4 hr | ― |

― =no change in FL

*All tubes were stored at -80°C

**FL values were evaluated at 1 week of subzero storage

^δ^Time of whole blood at 22°C before centrifugation and subzero storage
